# Awakening of SCHLAFEN 11 by immunohistochemistry: a new biomarker predicting response to chemotherapy

**DOI:** 10.1007/s00428-021-03051-3

**Published:** 2021-02-10

**Authors:** Reinhard Buettner

**Affiliations:** Institute for Pathology, University Hospital Cologne, Kerpener Str. 62, D-50937 Cologne, Germany

Developing predictive biomarkers indicating response or resistance to therapy paved the way for broad clinical implementation of personalized therapies by tyrosine kinase inhibitors [1] in many tumor types. In contrast, predicting response to chemotherapy by the widely used DNA-damaging agents including platinum derivates has been an unmet need since the early 1960s. Over the years, two pivotal DNA damage repair enzyme complexes have been identified: ERCC1 and SCHLAFEN 11 (SLFN11).

DNA excision repair protein ERCC1 together with ERCC4 forms the ERCC1-XPF enzyme complex that participates in DNA nucleotide excision repair (NER) and DNA recombination [2]. Measuring ERCC1 activity has potential clinical utility in predicting resistance to platinum as NER is the primary mechanism to remove platinum-DNA adducts from tumor DNA [3]. However, as the activity of the ERCC1-XPF enzyme complex in cancer cells is regulated on manifold levels including expression of different proteins and polymorphisms in codon 118, and may be affected by mutations and gene silencing, straight-forward and reliable assays have yet to be introduced into the clinical practice of pathologists. Thus, it seems that a more general marker of cell death induction after cytotoxic therapies with platinum agents, topoisomerase inhibitors (etoposide, doxorubicin), and replication inhibitors (cytarabine, gemcitabine) may be more suitable than assessing individual DNA repair pathways. Therefore, SLFN11 as a master regulator of cell death responses to DNA damage has gained significant interest.

**Fig. 1:**
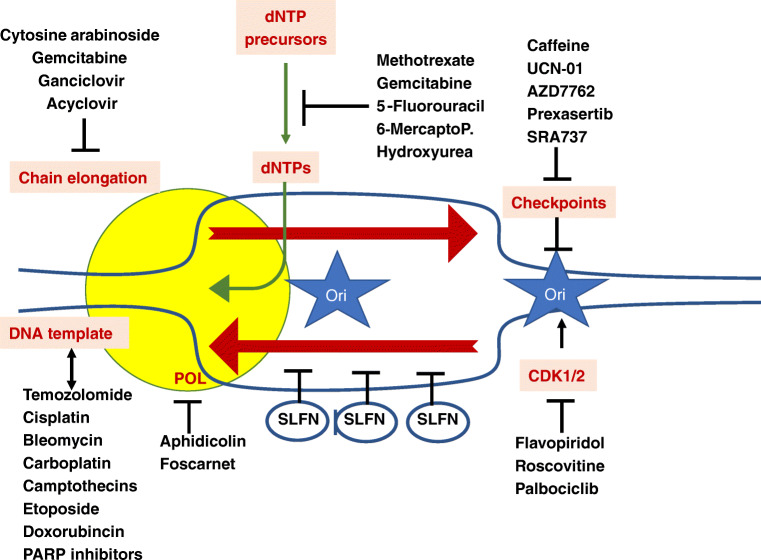
Different types of DNA damage leading to DNA-damage stressed replication fork progression stalled by SLFN11

In this context, the manuscript of Takashima and colleagues in this issue [4] provides a significant step forward with regard to assaying the DNA damage repair complex SLFN11. As an inhibitor of DNA replication, SLFN11 has been shown to trigger cell death in response to DNA damage from many different agents. This function involves ATR-independent blockade of DNA damage and stressed replication fork progression, possibly acting as a helicase and ultimately leading to cell death [5, 6]. The pivotal role of SLFN11 protein as the guardian of DNA-damaged cells is depicted in Fig. 1. Importantly, the non-redundant function of SFN11 has been shown in small cell lung cancer (SCLC), a clinically most relevant model of emerging chemoresistance. As practically all SCLCs initially respond well to combined cytotoxic chemotherapy with cisplatinum and etoposide, they irreversibly relapse with a chemo-resistant phenotype causing fatal outcome of the disease. Studies on the mechanism of acquired chemoresistance in SCLC indicated that Enhancer of Zeste 2 Polycomb Repressive Complex 2 (EZH2) mediates resistance via silencing and downregulation of the SLFN11 gene [7]. Thus, combining EZH2 inhibition with chemotherapy may be a novel concept avoiding resistance to chemotherapy.

In their landmark study, Takashima and colleagues used a reliable antibody and established a semi-quantitative scoring system of SLFN11 to assess SLFN11 expression in approximately 700 malignant tumors as well as in adjacent non-tumorous tissues across 16 different human adult organs. Thereby, the authors provide a reference repository of SLFN11 expression in human cancers and tissues. In addition, they assess the expression of SLFN11 in The Cancer Genome Atlas (TCGA) repository and unravel important discrepancies. Assessing SLFN11 by whole tissue-gene expression transcriptomes largely overrates SLFN11-negative tumors as TCGA samples are a mixture of tumor cells and infiltrating immune cells, including T cells, B cells, and macrophages, that have strong SLFN11 expression. In summary, the study of Takashima and colleagues in this issue will certainly trigger further studies of clinically annotated tumor samples and their extent of regression after chemotherapy. Ultimately, establishing such a clinically useful and easy assay of prediction of chemoresponse should also push forward concepts of neoadjuvant therapies. It is rewarding to see that immunohistochemistry, the good old working horse of diagnostic pathologist, leads the way to improve precision of chemotherapies and has a role even in times of large cancer genome and transcriptome studies.
